# Hepato-biliary stem cells: facts and fancies

**DOI:** 10.1186/2047-783X-19-S1-S5

**Published:** 2014-06-19

**Authors:** Neil D Theise

**Affiliations:** 1 Departments of Pathology and Medicine (Digestive Diseases) of Beth Israel Medical Center, Icahn School of Medicine at Mount Sinai, New York NY, 10003, USA

## The canals of Hering and stem cells

At the close of the 20^th^ century, a decades question long was finally settled: both rodent and human livers had facultative hepato-biliary stem cells. The stem cell niche in which these cells’ activities were identified was the canal of Hering (CoH) [1] – the link between hepatocyte canaliculi and the biliary tree. This structure had been *functionally* demonstrated by Ewald Hering in 1857, but its anatomic structure was merely *inferred*, *but not visualized*. Hering’s suggested that the link was located at the limiting plate, where hepatic parenchyma encountered portal tract stroma. His initial drawings depicted that hypothesis. The first direct imagings of the CoH were via electron microscopy, which confirmed that the CoH, as Hering intuited, was comprised of hepatocytes on one side and the smallest cholangiocytes on the other, but these high powered, ultrastructural views could not really explore the canal’s tissue level anatomic structure.

Immunohistochemistry (for biliary keratins) and serial sectioning to create a three dimensional analysis of CoH in human livers solved the question [2]. Human CoH were not as small as thought and not restricted to the limiting plate; rather, they were delicate structures that extended across the limiting plate, into the periportal parenchymal regions, as much as a third of the way toward the central vein (but no further). It was from these structures that new hepatocytes were seen to be arising in the setting of severe acute injury (e.g. acetaminophen toxicity) [2] and in chronic liver diseases of all kinds (reviewed in [3]).

These findings led to significant paradigm shifts. For hepato-biliary regeneration, settling of the argument as to whether there actually were liver stem cells potentiated explosive creativity in exploring their physiologic behavior and theraperutic potential. For pathology, the ubiquitous, but poorly understood “ductular reactions” (DR) – seen in all severe acute injuries and in all advanced stage chronic liver disease – were now understood to be tissue activations of the hepatobiliary stem cell niche [3].

## Identification of diverse hepatobiliary stem cell niches

The state of the art for identifying stem cell niches in any organ is called the “label retaining cell (LRC) assay” and exploits the definitional qualities of stem cells that are rarely dividing (“quiescent”) and productive of differentiating, rapidly proliferative, *transit amplifying*, progenitor cells (the hepatobiliary cells of DR in humans, the “oval cells” in rodents). Pulse labeling with a marker (e.g. BrdU) or with inducible transgenic markers can lead to labeling of nuclei of dividing stem and progenitor cells. Since transit amplifying progenitor cells are rapidly proliferative, the label is washed out. The other daughter cell restores a quiescent stem cell to the niche which thus, being quiescent, retains the label. One such study, to my knowledge, has been published for the liver, by my own group. Kuwahara et al [5], exploiting a murine model of acetaminophen toxicity], highlighted four possible stem cell niches all of which were predicted by other investigators in other models (see [4] for details):

• small cuboidal cells in the CoH;

• occasional cholangiocytes in the interlobular bile ducts;

• periductal null cells (devoid of epithelial or other stem cell markers); these were previously suggested by work from the Sell laboratory and/or could be related to multipotent stromal cells;

• peribiliary hepatocytes (PBH), i.e. hepatocytes that were directly adjacent to the CoH or bile ductules which were, to our surprise, the most commonly found LRC in this model.

Finally, in a research field too complex and robust to summarize here, there also appear to be extrahepatic stem/progenitor cells which may be, for example, circulating mesenchymal stem cells, hepatopoietic stem cells or so called “very small embryonic-like stem cells” some or all of which may derived from niches in the bone marrow. This field remains highly contentious: while there is significant (often political) opposition to these possibilities, there is also still greater support in the published evidence to date [5]. Time, of course, will tell.

## The puzzles of liver reconstitution & the unique hepatic microanatomy

One persistent question that remains, even as consensus regarding the existence of hepatobiliary stem cells has solidified, is why, unlike other organs, do these stem cells seem not to lead to significant parenchymal reconstitution? In all injury models in mice and in acute liver disease in humans, hepatocytes still seem to regenerate the majority of the hepatic parenchyma. Only in chronic human liver disease – and then only in the advanced (often cirrhotic) stages of disease progression – are significant patches of parenchyma restored from stem/progenitors [6,7]. Why is the liver different from almost every other epithelial compartment in the body?

We have suggested one explanation that connects the PBH LRC data in acetaminophen with the heretofore unremarked, unique aspects of basement membrane topography in the liver compared with other organs [8]. How might these relate? In all non-hepatic epithelial compartments of the body, basement membrane is continuous around all gland duct/acinar structures. Thus, when their stem cells produce transit amplifying progeny, the basement membrane provides a mechanical structure along which the cells can travel to their anatomical homes. In the liver, however, the basement membrane, while continuous around all biliary structures, stops and is interrupted at the CoH. The hepatocytes, that are directly linked to the CoH have no basement membrane, they and all the other hepatocytes are arrayed within the loosely aggregated collagen/reticulin fibers in the space of Disse. Thus, in response to injury, the repopulating hepatocytes have no guiding structure to lead them to reconnect to the cholangiocytes of the CoH.

One explanation to solve this puzzle is that the CoH secretes chemotactic factors which alert hepatocytes as to where to travel (though none have been recognized). Another possibility is that it is purely stochastic, regenerating hepatocytes filling the gap and eventually, randomly bumping into the CoH to re-attach. Either way, however, the hepatocytes not only have to get to the CoH, but once there have to turn on expression of the unique cell adhesion molecules that would allow for direct linking to these cholangiocytes, molecules that are very different from those that link hepatocytes to each other.

Our third hypothesis is intermediate between these two options [8]: that hepato-biliary stem cells are uniquely not tasked with tissue reconstitution. Instead, they are simply responsible for the re-establishment of the hepato-biliary link. The stem cell, having produced a hepatocytic neighbor, i.e. a PBH, has done its job. That PBH is already bound to its sibling cholangiocyte/stem cell in the CoH, but, being a hepatocyte, is also prepared to link to any hepatocyte that arrives next door through the stochastic movements of the repopulating hepatocytes. In human chronic liver disease, these PBH, eventually begin to contribute to significant repopulation because the older, previously repopulated hepatocytes become replicatively senescent. The PBH, having been largely quiescent over time; maintain the capacity to take over the repopulation.

We can also suggest that the basement membrane is therefore a key regulator of hepatobiliary stem cell functioning for regulating quiescent and differentiation. This may be related to adhesion molecules linking matrix proteins and cell membrane components, (e.g. integrins), but could also relate to the mechanical properties of basement membrane, such as stiffness. Testable predictions regarding this hypothesis, including these aspects, are currently under way.
